# The impact of selection criteria and study design on reported survival outcomes in extracorporeal oxygenation cardiopulmonary resuscitation (ECPR): a systematic review and meta-analysis

**DOI:** 10.1186/s13049-021-00956-5

**Published:** 2021-09-26

**Authors:** Sameer Karve, Dominique Lahood, Arne Diehl, Aidan Burrell, David H. Tian, Tim Southwood, Paul Forrest, Mark Dennis

**Affiliations:** 1 Sydney Medical School, University of Sydney, Sydney, NSW Australia; 2 Department of Cardiology, Royal Prince Alfred Hospital, Missenden Road, Camperdown, NSW 2050 Australia; 3 Intensive Care Service, Royal Prince Alfred Hospital, Sydney, NSW Australia; 4 Department of Anaesthesia, Royal Prince Alfred Hospital, Sydney, NSW Australia; 5 Australian and New Zealand Intensive Care Research Centre (ANZIC-RC), School of Public Health and Preventive Medicine, Monash University, Melbourne, VIC Australia; 6 Department of Intensive Care and Hyperbaric Medicine, The Alfred Hospital, Melbourne, VIC Australia; 7 Department of Anaesthesia and Perioperative Medicine, Westmead Hospital, Sydney, NSW Australia; 8 School of Medicine, University of Notre Dame, Sydney, NSW Australia

**Keywords:** Extracorporeal life support, Cardiology, Resuscitation and intensive care

## Abstract

**Background:**

The use of extracorporeal membrane oxygenation (ECMO) during cardiac arrest (ECPR) has increased exponentially. However, reported outcomes vary considerably due to differing study designs and selection criteria. This review assessed the impact of pre-defined selection criteria on ECPR survival.

**Methods:**

Systematic review applying PRISMA guidelines. We searched Medline, Embase, and Evidence-Based Medicine Reviews for RCTs and observational studies published from January 2000 to June 2021. Adult patients (> 12 years) receiving ECPR were included. Two investigators reviewed and extracted data on study design, number and type of inclusion criteria. Study quality was assessed using the Newcastle–Ottawa Scale (NOS). Outcomes included overall and neurologically favourable survival. Meta-analysis and meta-regression were performed.

**Results:**

67 studies were included: 14 prospective and 53 retrospective. No RCTs were identified at time of search. The number of inclusion criteria to select ECPR patients (*p* = 0.292) and study design (*p* = 0.962) was not associated with higher favourable neurological survival. However, amongst prospective studies, increased number of inclusion criteria was associated with improved outcomes in both OHCA and IHCA cohorts. (β = 0.12, *p* = 0.026) and arrest to ECMO flow time was predictive of survival. (β = -0.023, p < 0.001).

**Conclusions:**

Prospective studies showed number of selection criteria and, in particular, arrest to ECMO time were associated with significant improved survival. Well-designed prospective studies assessing the relative importance of criteria as well as larger efficacy studies are required to ensure appropriate application of what is a costly intervention.

**Supplementary Information:**

The online version contains supplementary material available at 10.1186/s13049-021-00956-5.

## Background

Extracorporeal membrane oxygenation (ECMO) for refractory cardiac arrest (RCA, ECPR) provides adequate systemic perfusion while the underlying cause may be diagnosed and treated. Whilst promising outcomes have been reported [1–3], survival rates vary widely [3] which is likely due to differences in study design, geographical location and inclusion criteria. Although a few well designed prospective studies exist [4], the use of ECPR has increased exponentially over the past decade [5].

Although there is evidence that ECPR is cost-effective [6–8] it is a resource-intensive and technically challenging support modality. Moreover, its cost-effectiveness is dependent on survival outcomes, which are influenced by inclusion criteria [9]. Current patient selection criteria for ECPR has largely been empirically determined [10].

We sought to analyse the survival rates in ECPR studies that had defined inclusion criteria for refractory out of hospital cardiac arrest (OHCA) and in hospital cardiac arrest (IHCA). Further, we sought to examine the association between the use of prospectively determined selection criteria (as opposed to retrospective no inclusion criteria) and the number of these inclusion criteria on reported survival outcomes.

## Materials and methods

### Protocol

This systematic review followed the Preferred Reporting Items for Systematic Reviews and Meta-Analyses (PRISMA) guidelines [11].

### Eligibility criteria

We used the PICO (Population, Intervention, Comparison, Outcome) search format: Among adults with cardiac arrest in any setting (OHCA or IHCA) who qualify for ECPR by inclusion and exclusion parameters outlined (P), does receiving ECPR, including ECMO or cardiopulmonary bypass, during cardiac arrest (I), compared to manual CPR and/or mechanical CPR (C) without any mechanical circulatory support, change meaningful outcomes (favourable neurological status or survival at study end) (O).

Contemporary studies from January 2000 to June 2021 were included. Only English language studies were included. Randomised trials, non-randomised controlled trials, and observational studies (cohort studies and case–control studies) were included. The population included adults (age above 12) with IHCA or OHCA of any origin. Animal studies, ecological studies, case reports, reviews, abstracts, editorials, comments, and letters to the editor were excluded. Studies with ≤ 5 patients receiving ECPR or studies that did not report the inclusion or exclusion criteria regarding the eligibility of ECPR or the timing of ECPR (i.e. during or after cardiac arrest) were excluded.

### Information sources and search strategy

We searched the following electronic bibliographic databases: Medline, Embase, and Evidence-Based Medicine Reviews (which includes the Cochrane Library). The search was repeated throughout to capture any articles published during the review process. We used a combination of various search terms for cardiac arrest and extracorporeal circulation. The bibliographies of included articles were reviewed for potential additional articles. The search terms, search strategies and results are shown in Additional file 1: Table 2.

### Study selection

Two reviewers (SK and DL), using pre-defined screening criteria, independently screened all titles and abstracts retrieved from the systematic review. Any disagreement regarding inclusion or exclusion was resolved via discussion between the reviewers and a third senior reviewer as needed. The third and senior reviewer reviewed all excluded titles and abstracts to ensure optimised sensitivity. Two reviewers then reviewed the full text-reports of all potentially relevant publications passing the first level of screening. Any disagreement regarding eligibility was resolved via discussion and adjudicated by the senior authors (PF and MD).

### Data collection and data items

Two reviewers using a pre-defined standardised data extraction form, extracted data as pertinent to the PICO. Data items included general study information (author, date, study design), cohort demographics (age, gender, comorbidities), arrest specifics (OHCA, IHCA, aetiology), inclusion criteria (total number, age, rhythm, time to ECMO flow, lactate, ETCO2) and outcomes (best Cerebral Performance Category (CPC) status, survival). Missing statistical parameters (i.e. odds ratios) of importance and variance measures (i.e. confidence intervals) were calculated if data permitted. Any discrepancies in the extracted data were identified and resolved with discussion and arbitration by the senior authors.

The primary outcome of interest was favourable neurological outcomes on discharge, using study-specified definitions. This typically included CPC status of 1–2, or Extended Glasgow Outcome Scores > 4. Secondary outcome included absolute survival irrespective of neurological outcome to the end of each study period.

### Risk of bias in individual studies

Two reviewers independently assessed the risk of bias in individual studies. The quality of studies was appraised using the Newcastle–Ottawa Scale (NOS) for assessing quality of non-randomised trials in meta-analysis. The NOS score is based on three domains including; patient selection, comparability and assessment of outcome or exposure. Scores of 0 to 9 were allocated to each study. Scores of 6 and above were deemed to be of high quality. Disagreements or discrepancies were resolved by consensus with discussion and review by a third senior reviewer.

### Data synthesis and statistical analysis

Studies were assessed for clinical (cohort demographics, inclusion criteria, interventions, and outcomes), methodological (study design or risk of bias), and statistical heterogeneity. Mean and standard deviation were calculated from median and interquartile range using the methods of Wan and colleagues [12]. Variables were aggregated with meta-analysis of proportions or means as appropriate, using a random-effects model to account for variations in study methodologies and heterogeneities. Meta-regression was performed to explore the effects of the number of inclusion criteria, the arrest to ECMO time, year of publication, type of study (prospective vs retrospective), on the heterogeneity of results. These moderating covariates were determined prior to analysis. Results are reported separately for pre-defined subgroups based on type of study (retrospective or prospective), and the location of cardiac arrest (IHCA vs OHCA). Additional sensitivity analyses of the effect of commonly utilised and clinically relevant inclusion criteria on neurologically intact survival rates was also assessed using meta-regression, with Bonferroni correction for multiple testing.

Evidence of publication bias was sought using the methods of Egger et al. and Begg et al. [13, 14] Contour-enhanced funnel plot was performed to aid in interpretation of the funnel plot.

All P values were 2-sided, with significance determined at less than 0.05. Statistical analyses were performed in R (version 4.0.2, R Foundation for Statistical Computing, Vienna, Austria).

## Results

### Study selection

**Fig. 1:**
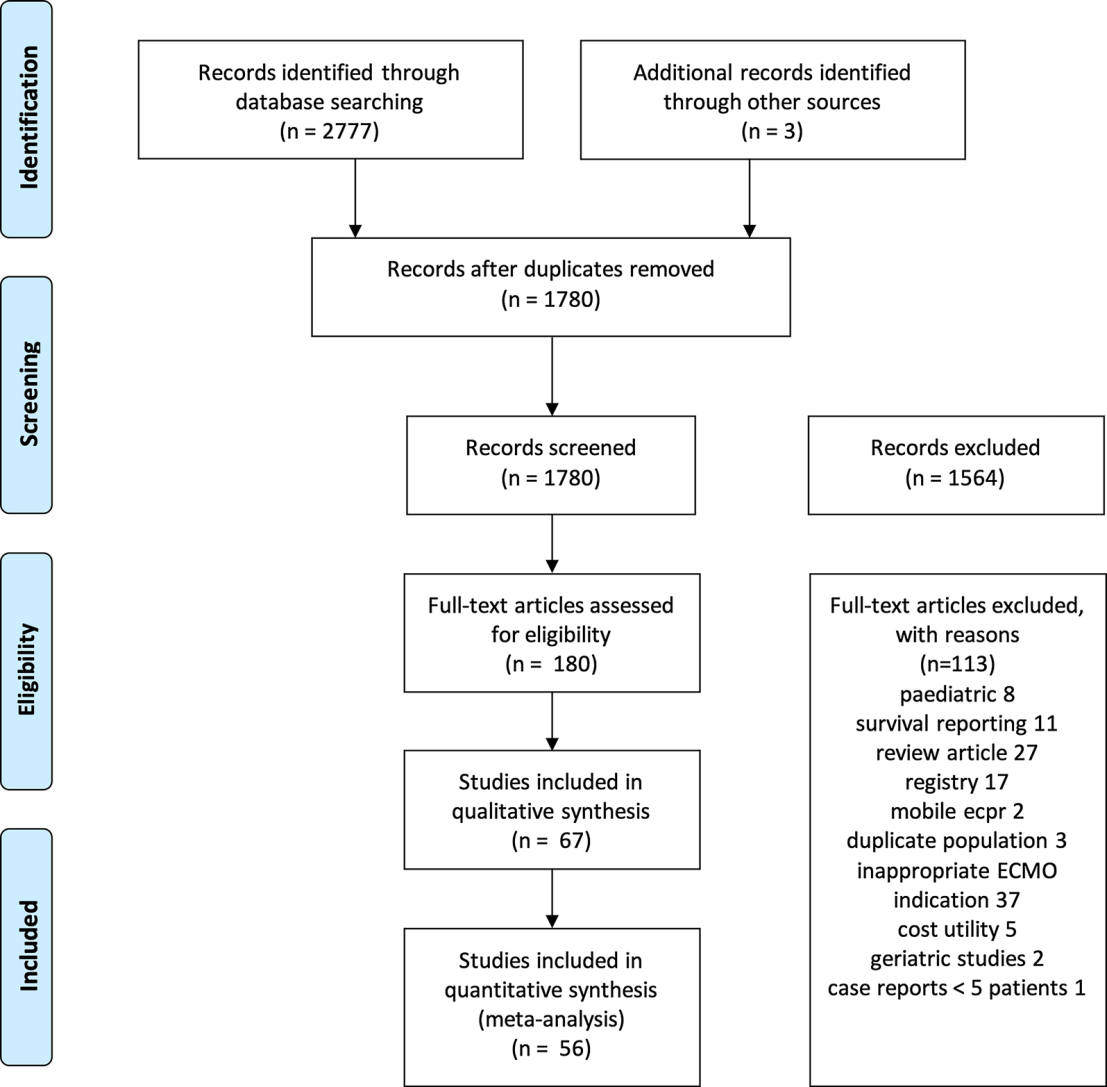
PRISMA flowchart

The search strategy identified 1780 unique records of which 180 records were eligible for full-text review. A PRISMA diagram of the study selection process is presented in Fig. 1. No randomised clinical trials were identified during the search. 67 observational studies (14 prospective and 53 retrospective studies) met all the inclusion criteria and none of the exclusion criteria. Each included study, cohort specifics, eligibility criteria and outcomes are provided in Additional file 1: Table 3.

**Fig. 2:**
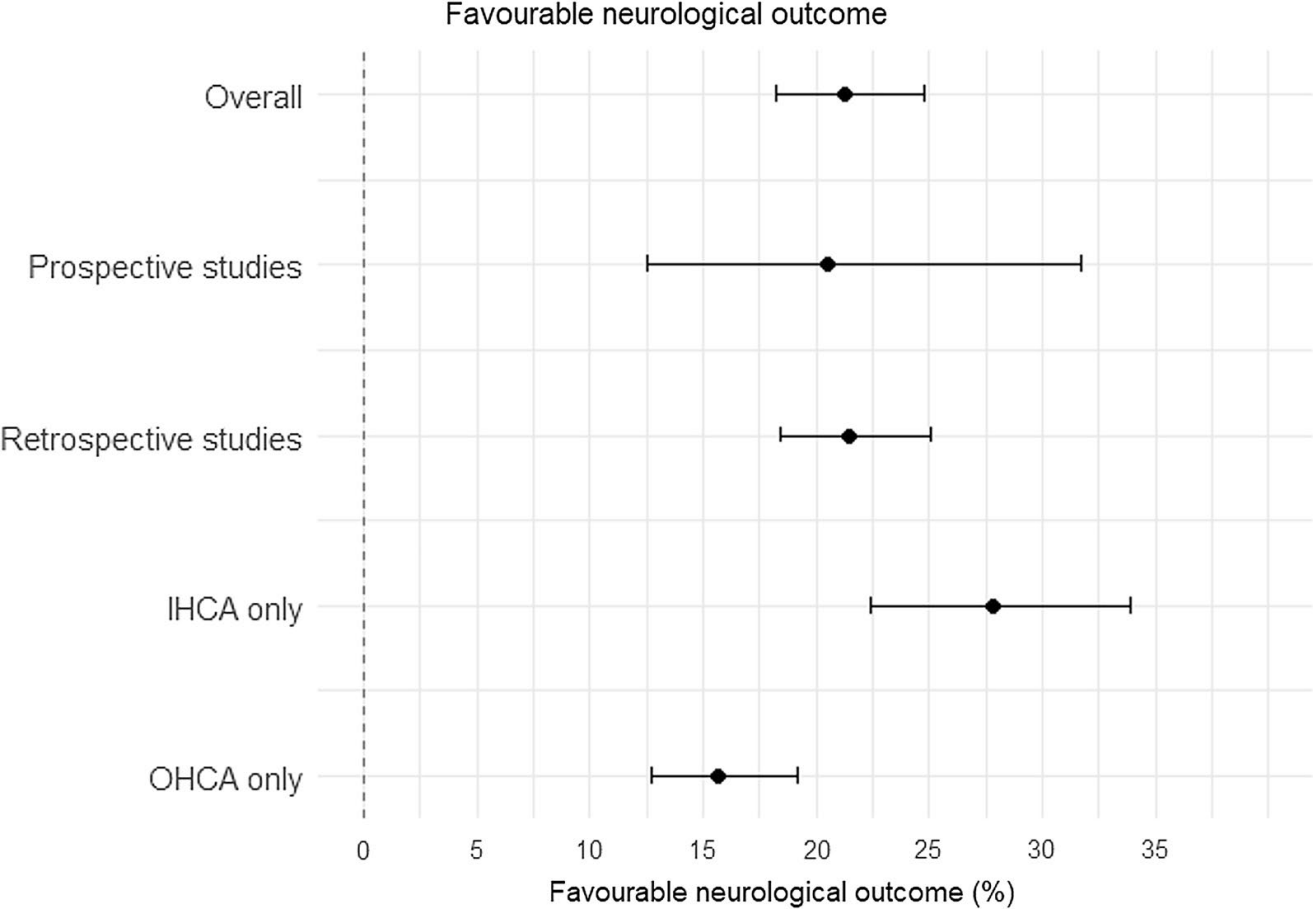
Simplified forest plot of favourable neurological outcome (CPC 1–2)

Neurologically survival rates are depicted in Fig. 2 and full Forrest Plot (Additional file 1: Fig. 1). The overall survival rate for all included studies was 28% (CI 24–33) and survival with favourable neurological outcome was 21% (CI 18–25), 16% (CI 13–19) in OHCA and 28% (CI 22–34) in IHCA (Table 1). The most frequent included criteria were age n = 43 (78%), absence of severe comorbidities n = 33 (63%), witnessed arrest n = 25 (47%) and bystander CPR n = 24 (45%). (Table 1 and list of frequencies of inclusion criteria in Additional file 1: Table 4).

**Table 1:** Cumulative study outcomes

Outcomes	Prospective	Retrospective	Overall
Favourable neurological outcome CPC1-2 (%)	21 (13–32)	22 (18–25)	21 (18–25)
IHCA only (%)	28 (17–42)	28 (22–35)	28 (22–34)
OHCA only (%)	18 (10–30)	15 (12–18)	16 (13–19)
Overall survival (%)	28 (13–50)	28 (24–32)	28 (24–33)

Results are aggregated using random-effects meta-analysis of proportions or means, as appropriate. Data is presented with 95% confidence interval of the pooled values in brackets. IHCA, in-hospital cardiac arrest; OHCA, out of hospital cardiac arrest

On meta regression, prospective study design not associated with higher neurological survival outcome (*p* = 0.962), nor was the presence of greater number of inclusion criteria (*p* = 0.292).

### Prospective studies

Fourteen studies were prospectively designed. Six included only OHCA cohort, two only IHCA cohort and six with a mixed cohort (OHCA and IHCA). Five were performed in Europe, four in Asia, three in North America and two in Australia. Years of patient recruitment ranged from 2004 to 2018.

The median age of the prospective cohort was 52 years (CI 48–55); 80% (CI 75–84) were male. The cause of arrest was cardiac in 85% (CI 75–92) of cases (Table 2). There was substantial heterogeneity in both the number and specific inclusion criteria reported (Table 2 with full detail in Additional file 1: Table 4).

**Table 2:** Summarised study characteristics

	Number of studies that reported characteristics (%)	Prospective median (95% CI)	Retrospective median (95% CI)	Overall median (95% CI)
*Demographics*				
Age*	63/67 (94%)	52 (48–55)	56 (55–58)	56 (54–58)
Male gender (%)	64/67 (95%)	80 (75–84)	74 (71–77)	75 (72–78)
Ischemic heart disease (%)	53/67 (79%)	51 (41–60)	53 (46–61)	53 (46–59)
Smoking (%)	24/67 (36%)	28 (21–38)	28 (25–33)	29 (25–33)
Diabetes (%)	47/67 (70%)	41 (14–75)	25 (21–30)	27 (21–34)
*Arrest*				
Witnessed arrest *(%)	51/67 (76%)	100 (95–100)	100 (37–100)	100 (96–100)
*Cardiac cause (%)	51/67 (76%)	85 (75–92)	90 (83–94)	89 (83–93)
Non-cardiac cause (%)	44/67 (65%)	17 (9–29)	13 (9–19)	14 (10–19)
Bystander CPR *(%)	40/67 (60%)	95 (68–99)	75 (57–87)	80 (66–90)
Shockable rhythm* (%)	56/67 (84%)	70 (51–84)	47 (39–56)	52 (45–60)
Non-shockable rhythm (%)	45/67 (67%)	41 (31–52)	56 (43–69)	52 (41–63)
Time to ECMO (min)	55/67 (82%)	74 (55–94)	57 (53–61)	60 (56–64)

Results are aggregated using random-effects meta-analysis of proportions or means, as appropriate. Data is presented with 95% confidence interval of the pooled values in brackets. CPR, cardiopulmonary resuscitation; ECMO, extracorporeal membrane oxygenation; IHD, ischemic heart disease

* Represent inclusion criteria commonly used for ECMO CPR

Common criteria included age 84% (CI 48–55), witnessed arrest 99% (CI 95–100), bystander CPR 95% (CI 68–99), shockable rhythm 70% (CI 51–84) and time to ECMO 74 min (CI 55–94). Less commonly serum lactate and ETCO_2_ were included 15% of prospective study criteria and signs of life was included in 7% of prospective study criteria. All studies reported survival to hospital discharge and most studies defined favourable neurological outcome as a (CPC) score of 1–2.

**Fig. 3:**
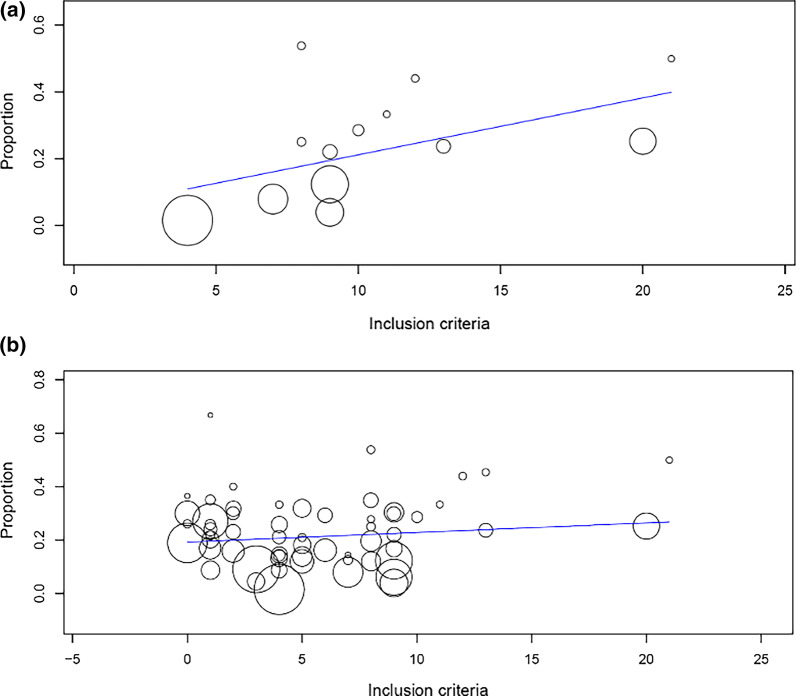
Analyses of proportion of favourable neurological outcomes against number of inclusion criteria. **a** Proportion of neurologically favourable outcomes against the number of inclusion criteria in prospective studies. **b** Proportion of neurologically favourable outcomes against the number of inclusion criteria in retrospective studies

The overall survival rate in the prospective studies was 28% (CI 13–50) and survival with favourable neurological outcome 21% (CI 13–32); 18% (CI 10–30) in OHCA and 28% (CI 17–42) IHCA (Table 1). On meta-regression of prospective studies only, a higher number of inclusion criteria was correlated with favourable neurological outcomes (β = 0.12, *p* = 0.026). As the number of inclusion criteria increased so too the proportion of favourable neurological outcomes. (Fig. 3a).

### Retrospective studies

Fifty-three studies were retrospective. Seventeen included only OHCA, eleven only IHCA and twenty-four were mixed OHCA and IHCA cohort and one unclassified [15]. A majority were performed in Asia and Europe with four conducted in Australia, and six in North America. Years of patient inclusion ranged from 2003 to 2019.

Retrospective study characteristics are outlined in Table 2. The mean age of the retrospective cohort was 56 years (CI 55–58); 74% (CI 71–77) were of male gender. The cause of arrest was cardiac in 90% (83–94) of cases. There was marked heterogeneity in both the number and specific inclusion criteria reported within studies, common criteria included age 73% (CI 55–58), witnessed arrest 100% (CI 37–100), bystander CPR 75% (CI 57–87), shockable rhythm 47% (CI 39–56) and time to ECMO 57 min (CI 53–61). Less commonly serum lactate was reported in 2% of retrospective study criteria and ETCO_2_ was reported in 12% of retrospective study criteria. (Additional file 1: Table 4) All studies reported survival to hospital discharge and most studies defined favourable neurological outcome as a CPC score of 1–2.

The overall survival rate in retrospective studies was 28% (CI 24–32); favourable neurological outcome occurred in 22% (CI 18–25) amongst all patients (OHCA and IHCA), 15% (CI 12–18) in OHCA and 28% (CI 22–35) in IHCA (Table 1). On meta-regression of these retrospective studies, the number of inclusion criteria and any specific criteria did not have an impact on favourable neurological outcomes (*p* = 0.699) (Fig. 3b).

### OHCA studies only

For OHCA patients (individual studies with only OHCA patients, or subpopulations of OHCA patients in mixed cohort studies), the overall survival rate was 16% (CI 13–19), 18% (CI 10–30) in prospective studies and 15% (CI 12–18) in retrospective studies. The number of inclusion criteria had no influence on outcomes (*p* = 0.134) nor did the type of study design (retrospective vs prospective, *p* = 0.534).

### IHCA studies only

For IHCA patients (individual studies with only IHCA patients, or subpopulations of IHCA patients in mixed cohort studies), the overall survival rate was 28% (CI 22–34); 28% (CI 13–50) in prospective studies and 28% (CI 24–32) retrospective studies. The type of study design had no influence on favourable outcomes (*p* = 0.992). The number of inclusion criteria had a non-significant trend towards favourable outcomes (*p* = 0.059).

### Risk of bias for individual studies

The risk of bias within individual studies was deemed acceptable for all studies. Three studies scored 5/9 on the NOS and the remainder scored 6/9. Use of Begg’s statistic (*p* = 0.298) and Egger’s regression intercept (*p* = 0.348) did not identify publication bias.

### Sensitivity analyses of individual inclusion criteria

Age, initial rhythm, shockable rhythm, bystander CPR, lactate and End-tidal CO_2_ were not found to be associated with favourable neurological outcome—Table 3. Signs of life (*p* = 0.03) and witnessed cardiac arrest (*p* = 0.04) showed a trend towards improved neurological outcomes, however both failed to reach the Bonferroni-corrected p-value threshold. Heterogeneities in definitions and reporting frequencies prevented more robust analysis of the effects of these individual inclusion criteria.

**Table 3:** Contribution of inclusion criteria on survival

Specific criteria	OR (95% CI)	*P* value
Age	1.05 (0.67 -1.66)	0.83
Initial rhythm	1.44 (0.93 -2.23)	0.1
Shockable rhythm	1.13 (0.75 -1.70)	0.57
Bystander CPR	0.96 (0.63 -1.48)	0.87
Lactate	1.14 (0.38 -3.37)	0.82
End-tidal CO_2_	0.68 (0.41 -1.10)	0.12
Signs of life	0.51 (0.28 -0.94)	0.03
Witnessed arrest	0.68 (0.48 -0.98)	0.04

## Discussion

We performed a comprehensive systematic review and meta-analysis of ECPR studies to assess the impact of study design and the number of inclusion criteria on outcome with additional sensitivity analyses of individual inclusion criteria on outcome.

We found relatively few prospective studies on its efficacy and no significant difference in either overall survival or survival with favourable neurological outcome between prospective and retrospective studies. Moreover, the number of inclusion criteria for ECPR did not correlate with an improvement in neurological outcomes for the total study population. When only prospective studies were analysed, the number of inclusion criteria was found to be associated with improved survival, as did the time from arrest to ECMO flow (low-flow time). Study heterogeneity of inclusion criteria prevented meaningful analyses of the impact of individual selection criteria on clinical outcomes.

Our finding that overall survival was similar between retrospective and prospective ECPR studies is likely due to a selection bias in the retrospective studies, i.e., patients with better prognostic markers are more likely to be offered ECPR for RCA [5]. Moreover, given the small number of prospective OHCA ECPR studies, survival rates are prone to influence by outlier results or variations in practice. For example, on sensitivity analysis, the removal of a single OHCA study with a very long arrest to ECMO flow time (> 70 min) [16] increased overall survival from 31.2% to 37.2%. Geographic variability in Emergency Medical Services (EMS) protocols (including delivery of conventional or mechanical CPR), transportation times and termination of resuscitation protocols are known to impact reported survival rates [17].

At present, there are no standardised or consensus ECPR eligibility criteria [18], consistent with marked heterogeneity of inclusion criteria we found in our study. Inclusion criteria that were consistently reported included: age, comorbidity, witnessed arrest with bystander CPR and initial shockable rhythm. These criteria have been largely based on the positive prognostic markers for conventional treatment (CCPR) of cardiac arrests [19]. Whilst these criteria have been shown to have prognostic value in individual ECPR studies [1], their relative contributions to clinical outcomes are yet to be tested in large ECPR cohorts. In a systematic review of ECPR for OHCA, factors associated with improved outcomes were low flow duration, shockable rhythm, lower lactate and higher pH levels [19] These factors were also important prognostic markers in ECPR for IHCA [20]. In our analysis, we were unable to determine the relative importance of most of these prognostic markers due to the variable inclusion criteria that were reported. However, in common with other studies [19–21] we found that shorter arrest to ECMO flow time (low flow time) was associated with improved outcomes. The question as to whether a shorter low flow time, may obviate the need of other inclusion criteria without impacting on survival outcomes is yet to be tested. Moreover, further studies into the relative weighting (or effect) of specific inclusion criteria are required. Of note more novel inclusion criteria, including end tidal CO_2_ [22] intermittent return of spontaneous circulation (ROSC) and signs of life [23] may have utility in refining patient selection but are only beginning to be explored prospectively in ECPR arrests and were very infrequently reported in studies in our analysis.

On pre-defined subgroup analysis prospective studies, we found that an increased number of selection criteria was associated with improved survival from ECPR. This finding supports the use of selective inclusion criteria in these patients, which are also associated with improved neurological outcomes in survivors [24, 25]. These results were driven by the Minnesota Resuscitation Consortium, which reported a survival rate of approximately 50% in refractory OHCA cases that met strict inclusion and exclusion criteria [1].

Whilst the application of more stringent inclusion criteria seems likely to improve survival from ECPR, fewer RCA patients will meet these criteria and be ECPR “eligible”. Currently, it is estimated that only 5–10% of OHCA cases meet basic ECPR eligibility criteria [26, 27]. The application of more stringent inclusion criteria will reduce this further potentially making ECPR, in some locations, a very infrequent event. Accurate data on which and how many patients are likely to benefit from ECPR is essential to ensure that these complex, expensive and low-volume programs deliver outcomes that remain at or above proven thresholds for cost-effectiveness [6, 7].

Despite considerable research and resource allocation over the past decade, ECPR studies have been predominantly observational to date [4]. Of the 67 studies in our analysis, only 14 were prospective, which limited the strength of our statistical analysis. This small number of studies is unlikely to ameliorate unmeasured confounders and there remains an obvious need for larger randomised studies to further examine efficacy and prognostic variables. Subsequent to our analysis, the first randomised trial of ECPR (the ARREST trial) for OHCA was published, [28] and the Prague-OHCA Study – NCT01511666 (presented in abstract), reported encouraging results in a ECPR patients with strict inclusion criteria. Whilst, the generalisability of these results and inclusion criteria, remains to be seen, these trials will undoubtedly further drive ECPR expansion.

Our study emphasises the importance of the prospective assessment of selection criteria when designing and comparing these programs but also the limitations of current data in analysing patient selection criteria. Further data is essential to ensure the rational development of an expensive and challenging, but promising application of this technology.

### Limitations

The evidence base was found to be observational in nature with marked heterogeneity in inclusion and exclusion criteria as well as variability in reported results. Furthermore, survival outcomes were missing from six retrospective studies limiting overall analysis. Arrest characteristics such as bystander CPR were not always reported, how this affects low flow time is questionable. We did not review complications associated with ECPR and how these may affect survival.

## Conclusions

In this review, study design was not associated with improved outcomes. Within prospectively designed studies, more stringent selection criteria and specifically arrest to ECMO time, likely improves outcomes. Significant study heterogeneity and a small number of prospectively designed studies limited statistical inference. Large well-designed prospective studies that enable assessment ECPR prognostic variables, inclusion criteria and their relative weighting of effects on outcomes are urgently required to ensure optimal patient selection for ECPR.

## Supplementary Information

**Additional file 1.Table 1**. Literature search strategy for MEDLINE via Pubmed. **Table 2**. Risk of bias assessment. **Table 3**. Study Selection and Characteristics. **Table 4**. Inclusion criteria frequency in prospective and retrospective studies. **Figure 1**. Study specific forest plot of main findings.

## Data Availability

The data underlying this article are available in the article and in its online supplementary material.

## Abbreviations

CPC: Cerebral performance category
CPR: Cardiopulmonary resuscitation
ECMO: Extracorporeal membrane oxygenation
ECPR: Extracorporeal cardiopulmonary resuscitation
ETCO2: End tidal carbon dioxide
IHCA: In-hospital cardiac arrest
OHCA: Out of hospital cardiac arrest
PRISMA: Preferred reporting items for systematic reviews and meta-analyses
NOS: Newcastle–Ottawa scale
PICO: Population intervention comparison and outcome
RCA: Refractory cardiac arrest
RCT: Randomised Controlled Trial
