# Supplementary material for: The impact of selection criteria and study design on reported survival outcomes in extracorporeal oxygenation cardiopulmonary resuscitation (ECPR): a systematic review and meta-analysis
Source: Scand J Trauma Resusc Emerg Med. 2021 Sep 26;29:142. doi: 10.1186/s13049-021-00956-5 (PMC8474891; doi:10.1186/s13049-021-00956-5)
Supplement: Supplementary file 1 — Additional file 1.Table 1. Literature search strategy for MEDLINE via Pubmed. Table 2. Risk of bias assessment. Table 3. Study Selection and Characteristics. Table 4. Inclusion criteria frequency in prospective and retrospective studies. Figure 1. Study specific forest plot of main findings. [file 13049_2021_956_MOESM1_ESM.docx]

**Supplementary Table 1 .** Literature search strategy for MEDLINE via Pubmed.

Date range: from January 2000 to September 2016, limited to Humans

Date searched: 2016.09.23

| #1 | "Extracorporeal Membrane Oxygenation"[Mesh] OR "extra-corporeal membrane oxygenation"[TW] | 4,724 |
| --- | --- | --- |
| #2 | "Extracorporeal Circulation"[Mesh] OR "Extra-corporeal Circulation" [TW] | 23,719 |
| #3 | "extracorporeal life support"[TW] OR "extra-corporeal life support"[TW] | 838 |
| #4 | "extracorporeal cardiopulmonary resuscitation"[TW] OR "extra-corporeal cardiopulmonary resuscitation"[TW] | 112 |
| #5 | "ECMO"[TW] OR "E-CMO"[TW] | 2,703 |
| #6 | "ECLS"[TW] OR "E-CLS"[TW] | 485 |
| #7 | "ECPR" OR "E-CPR" | 91 |
| #8 | #1 OR #2 OR #3 OR #4 OR #5 OR #6 OR #7 | 24,399 |
| #9 | "Heart Arrest"[Mesh] | 21,963 |
| #10 | "Out-of-Hospital Cardiac Arrest"[Mesh] | 1,954 |
| #11 | “cardiac arrest”[TW] | 12,843 |
| #12 | “OHCA”[TW] | 889 |
| #13 | #9 OR #10 OR #11 OR #12 | 26,082 |
| #14 | #8 AND #13 | 812 |

Repeat search June 2020

**Supplementary Table 2.** Risk of bias assessment

| Author, year | Study participation | Study attrition | Predictor measurement | Outcome measurement | Confounding | Analysis and reporting |
| --- | --- | --- | --- | --- | --- | --- |
| Belle, 2012 | ● | ● | ● | ● | ● | ● |
| Debaty, 2010 | ● | ● | ● | ● | ● | ● |
| Fagnoul, 2013 | ● | ● | ● | ● | ● | ● |
| Ha, 2016 | ● | ● | ● | ● | ● | ● |
| Kagawa, 2010 | ● | ● | ● | ● | ● | ● |
| Kim, 2014 | ● | ● | ● | ● | ● | ● |
| Le Guen, 2011 | ● | ● | ● | ● | ● | ● |
| Leick, 2013 | ● | ● | ● | ● | ● | ● |
| Maekawa, 2013 | ● | ● | ● | ● | ● | ● |
| Mégarbane, 2011 | ● | ● | ● | ● | ● | ● |
| Mochizuki, 2014 | ● | ● | ● | ● | ● | ● |
| Nagao, 2010 | ● | ● | ● | ● | ● | ● |
| Tanno, 2008 | ● | ● | ● | ● | ● | ● |
| Wallmüller, 2013 | ● | ● | ● | ● | ● | ● |
| Yannopoulos, 2016 | ● | ● | ● | ● | ● | ● |

The risk of bias was rated as low (●), moderate (●), or high (●) for each domain, using the refined Quality in Prognosis Studies (QUIPS) tool.

Supplementary Table 3– Study Selection and Characteristics

| Author, country and year | Total (n) | Age, | Gender (male) | Ischaemic aetiology | Cardiac aetiology | Witnessed | Bystander | Shockable rhythm | Time to ECMO (min) | Favourable neurological outcome (n) | Inclusion | Exclusion |
| --- | --- | --- | --- | --- | --- | --- | --- | --- | --- | --- | --- | --- |
| Prospective | | | | | | | | | | | | |
| Bartos, USA, 2018 1 | 83 OHCA | 56 | 64 | NR | NR | 60 | 60 | 83 | 72.3 | NR | N= 6 | N=4 |
| Chen, Taiwan, 200818 | 59 IHCA | 57.4  (12.5) | 50 | 37 | 59 | 59 | NR | 29 | 52.8 | 14 | N=4 | N= 9 |
| Chonde, USA, 202019 | 5 OHCA | 47 (32-  53) | 4 | 2 | 5 | 5 | 5 | 5 | 34 (30-45)  arrival to hospital and 21(16-33)  arrival at ED to cannulation | NR | N=4 | N =0 |
| Dennis, Australia, 202020 | 25  14 IHCA  11 OHCA | 57 (39-  65) | 17 | 12 | 21 | 25 | 25 | 18 | 57 (38-73) | IHCA = 8  OHCA = 3 | N=9 | N=3 |
| Fagnoul,  Belgium, 201321 | 24  10 IHCA  14 OHCA | 48 (38-  55) | 14 | 7 | 15 | 24 | 22 | 10 | 58 (45-70) | IHCA = 3  OHCA = 3 | N=5 | N= 3 |
| Lamhaut, France, 2017, period  115 | Period 1: 114 OHCA | 50.6 | 91 | NR | NR | 114 | Y | 56 | No flow: 3.7 minutes  No flow >/= 5 minutes in 36 patients  Low flow: 93 minutes | 9 | N=7 | N=0 |
| Lamhaut, France, 2017, period  215 | Period 2: 42 OHCA | 53.8 | 37 | NR | NR | 42 | Y | 25 | No flow: 2.5 No flow > 5 minutes:  9  Low flow: 70.9 minutes | 12 | N=10 | N= 0 |
| Le Guen, France, 201122 | 51 OHCA | 42 +/-  15 | 46 | 11 | NR | 51 | NR | 32 | 120 (102-149) | 2 | N=5 | N=4 |
| Lin, Taiwan, 201023 | 59 IHCA | 59  (11.2) | 47 | 36 | 54 | 55 | NR | 28 | NR | 13 | N=3 | N=6 |
| Megarbane,  France, 201124 | 66  19 IHCA  47 OHCA | NR | NR | NR | Y | 66 | NR | Yes but  number not reported | 155 (120-180) | IHCA = 1  OHCA = 0 | N=2 | N=2 |
| Roncon- Albuquerque, Portugal,  201825 | 18  13 IHCA  5 OHCA | 52 (45-  56) | 11 | 13 | 14 | 18 | 18 | 9 | 40 (35-55) | OHCA = 2  IHCA = 4 | N=9 | N=2 |

| Sakamoto, Japan, 201426 | 260 OHCA | 56.3 | 235 | 165 | 226 | 186 | 127 | NR | 45-60 | 32 | N=4 | N=5 |
| --- | --- | --- | --- | --- | --- | --- | --- | --- | --- | --- | --- | --- |
| Stub, Australia, 201527 | 26  15 IHCA  11 OHCA | 52 (38-  60) | 20 | 14 | 21 | NR | 26 | 19 | 50 (40-85) | OHCA = 5  IHCA = 9 | N=5 | N=3 |
| Wang, Taiwan, 2014 OHCA28 | 230  31 OHCA | 50.7  +/- 15.3 | 23 | 19 | 24 | 31 | NR | 15 | 67.5 +/- 30.6 | OHCA = 8 | N=6 | N=14 |
| Wang, Taiwan, 2014 IHCA | 199 IHCA | 55.7  +/- 15.1 | 155 | 85 | 133 | 199 | NR | 91 | 44.4 +/- 24.7 | IHCA = 50 |  |  |
| Yannopoulos  , USA, 201629 | 18 OHCA | 56 | 14 | NR | NR | 11 | 12 | 18 | 66.4 +/- 9 | 9 | N=13 | N=8 |
| Retrospective | | | | | | | | | | | | |
| Avalli, Italy, 201130  IHCA | 24 IHCA | 67 (61-  73) | 16 | 9 | 22 | 24 | 24 | 12 | No Flow: 1  (0.5-1)  Low flow: 55  (40-70) | 9 | N=4 | N=5 |
| Avalli, Italy, 201130  OHCA | 18 OHCA | 46 (37-  64) | 17 | 12 | 15 | 18 | 10 | 16 | No flow: 1(1-  7.25)  Low flow 77  (69-101) | 1 | - | - |
| Axtell, USA, 202031 | 54 OHCA | 58(46-  65) | 40 | 33 | 45 | 42 | NR | 25 | NR | 13 | N=1 | N=6 |
| Bednarcyzk, Canada,  201432 | 22 IHCA | NR | NR | NR | NR | 52 | NR | NR | Low flow: 48.78 +/- 21 | 10 | N=4 | N=9 |
| Bellezo, USA, 201233 | 18 OHCA | 55.7  +/- 12.8 | 14 | 11 | 15 | NR | NR | NR | NR inclusion criteria states less than 60 minutes | 5 | N=2 | N=6 |
| Blumenstein, Germany, 2016, 34 | 52 IHCA | 72 (55-  77.9) | 28 | 23 | NR | 52 | NR | 1 | NR | 11 | N=4 | N=7 |
| Casadio, Italy, 201735 | 112  39 IHCA  73 OHCA | 57.8  +/- 11.1 | 92 | 83 | 112 | 112 | 99 | 93 | NR | 83% of 30  (21.6) | N=8 | N=3 |
| Choi, Korea, 201636 | 320 OHCA | 56 (45-  65) | 258 | 85 | 320 | 228 | 95 | 93 | NR | 29 | N=3 | N=2 |
| Chou, Taiwan, 201437 | 43 IHCA | 60.5  +/- 11.6 | 40 | 43 | 43 | NR | NR | 26 | NR | NR | N=3 | N=5 |
| Daou, France, 202038 | 113  60 IHCA  53 OHCA | 55 (46-  63) | 85 | 61 | 78 | 113 | 113 | 8 | 84 (55-122) | 7 ihca 11 OHCA | N=2 | N=5 |

| Dennis, Australia, 201739 | 37  25 IHCA  12 OHCA | 54 (47-  58) | 27 | 11 | 19 | 27 | 30 | 19 | 45 (30-70) | 9 ihca, 4 ohca | N=1 | N=1 |
| --- | --- | --- | --- | --- | --- | --- | --- | --- | --- | --- | --- | --- |
| Ellouze, France, 201840 | 65  43 IHCA  22 OHCA | 56 (43-  65) | 45 | NR | 48 | NR | 65 | 22 | IHCA: 60 (45-  89)  OHCA: 90 (66-  138) | 9 ihca, 6 ohca | N=2 | N=6 |
| Fjølner, Denmark, 201641 | 21 OHCA | 56 (19-  73) | 12 | 13 | 21 | 21 | 21 | 9 | 121 (55-192) | 7 | N=4 | N=4 |
| Grunau,  Canada, 201642 | 1206 OHCA | 55 (47-  60) | 908 | NR | NR | 960 | 622 | NR | NR | NR | N=4 | N=1 |
| Ha, Korea, 201743 | 35 OHCA | NR | 29 | 12 | 26 | 29 | 17 | 18 | Survivors: 72  (57-91.5)  Non survivors: 82 (65.3-104.8) | 9 | N=1 | N=6 |
| Han, Korea, 201944 | 100  75 OHCA  25 IHCA | Surviv ors: 40  +/- 15 | 12 | NR | 14 | 13 | 12 | 10 | 64 +/- 24.5 | 12 | N=5 | N=4 |
|  |  | Non- survivo rs 58  +/- 14 | 62 | NR | 75 | 73 | 61 | 44 | 76 +/- 20.1 | - | - | - |
| Haneya, Germany, 201245 | 85  26 OHCA  59 IHCA | 57 (47-  73) | 61 | 38 | 54 | NR | NR | 25 | 51 +/- 35  (40:20-70) | 27 | N=2 | N=4 |
| Jo, Korea, 201146 | 83 IHCA | 58.1  +/- 17.3 | 40 | NR | 68 | NR | NR | 39 | 37.2 +/- 26,  median and IQR : 30 (18-  60) | 29 | N=3 | N=5 |
| Jung, Germany, 201647 | 117  83 IHCA  34 OHCA | 61  (51;74) | 80 | 86 | 74 | NR | NR | 74 | NR | 17 | N=4 | N=2 |
| Kagawa,  IHCA Japan, 201048 | 38 IHCA | 68 (58-  73) | 22 | NR | 31 | 35 | 35 | 10 | 25 (21-43) | 10 | N=5 | N=2 |
| Kagawa OHCA48 | 39 OHCA | 56 (49-  64) | 33 | NR | 37 | 32 | 28 | 19 | 49 (45-65) | 4 | - | - |
| Ko, Korea, 202049 | 42 IHCA | Asystol e 62.5  (47-74) | 24 | 18 | NR | NR | NR | 0 | 36.5 (20-55) | NR | N=2  -  - | N=6  -  - |
|  | 163 IHCA | PEA 67  (56.5-  75) | 102 | 76 | NR | NR | NR | 0 | 28 (18-43.5) | NR |  |  |
|  | 89 IHCA | Shock able rhythm 59 (51-  68) | 72 | 52 | NR | NR | NR | 89 | 35 (22-51) | NR |  |  |

| Kim, Korea, 201450 | 55 OHCA | 53(41-  68) | 41 | 41 | 49 | 43 | 23 | 31 | Low flow: 7 (0-  13)  No flow: 62  (47-89( | 8 | N=4 | N=4 |
| --- | --- | --- | --- | --- | --- | --- | --- | --- | --- | --- | --- | --- |
| Komeyama, Japan, 201951 | 67  51 IHCA  16 OHCA | 62.35  +/- 13.5 | 14 | 19 | 67 | 67 | NR | 40 | Favourable: 37.8 +/- 28.1 | 17 IHCA and 3 OHCA | N=4 | N=6 |
|  |  | 65.11  +/- 14.32 | 36 | 38 | - | - | - | - | Unfavourable: 53/6 +/- 30.7 | - |  |  |
| Kuroki, Japan,  201652 | 119  82 IHCA  37 OHCA | 63.2  +/- 11.8 | 108 | 73 | 119 | NR | NR | 39 | 34.2 +/- 16.1 | 38 | N=5 | N=0 |
| Lazzeri, Italy, 201353 | 16 IHCA | 54.8  +/- 9 | 12 | 10 | 16 | NR | NR | NR | Low flow: 51.9  +/- 24.8 | 2 | N=4 | N=3 |
| Lee, Korea, 201654 | 23 OHCA | 55  (40,68) | 20 | 20 | NR | 23 | 14 | 20 | Collapse to ECMO: 84  (61,101)  Low flow time: 62(53,86) | 5 | N=1 | N=3 |
| Lee Weekday, Korea, 201655 | 135 IHCA | 65.5  (53-75) | 80 | 61 | 97 | 16 | NR | 37 | 31 (20-52) | 41 | N=3 | N=6 |
| Lee, weekend, Korea, 201655 | 65 IHCA | 59 (47-  70) | 41 | 28 | 45 | NR | NR | 19 | 47 (30-65) | 11 |  |  |
| Leick, Germany, 201356 | 28 OHCA | Non survivo r 53.9  +/- 15.9  survivo r 60.3  +/- 9.6 | Non survivors 10  Survivors 5 | Non survivors 10,  survivors 5 | 27 | 28 | NR | NR | NR | 4 | N=4 | N=5 |
| Liu, Taiwan, 201157 | 11  1 OHCA  10 IHCA | 55.9  (7.6) | 9 | 9 | 11 | 10 | 374 | 4 | 53 (40-61.3) | 4 | N=0 | N=7 |
| Lunz, Europe, 202011 | 423  163 IHCA  258 OHCA | 57 (48-  65) | 330 | 330 | 116 | 307 | 9 | NR | 44 (31-45) | 56 IHCA, 24 OHCA | 0 | 0 |
| Maekawa, Japan, 201358 | 53 OHCA | 54 (47-  60) | 44 | 3 | NR | 6 | NR | 32 | No flow 2(0-8)  Low flow 49  (41-59) | 8 | N=6 | N=2 |
| Mandigers, Netherlands, 201959 | 19 13 OHCA  6 IHCA | 40 (30-  60) | 8 | 8 | 19 | 19 | NR | NR | NR | 4 | N=5 | N=0 |

| Mazzeffi, France, 201660 | 23 IHCA | 57 +/-  15 | 14 | 14 | 11 | NR | 7 | 6 | 67 (45-85) | 6 | N =0 | N=0 |
| --- | --- | --- | --- | --- | --- | --- | --- | --- | --- | --- | --- | --- |
| Otani, Japan, 201661 | 40 OHCA | 58 (49-  72) | 39 | 25 | 34 | 40 | 10 | NR | 77 (39-98) | NR | N=2 | N=0 |
| Pang, Singapore, 201762 | 79  73 IHCA  6 OHCA | 49.9  +/- 12.4 | 62 | 62 | 79 | NR | 77 | 33 | NR | 16 | N=1 | N=5 |
| Park, Korea, 2014  Non- survivor63 | 152 IHCA | 61.5  +/- 16.4 | 66 | 20 | 83 | 104 | NR | 24 | 46 +/- 26 | - | N=1 | N=8 |
| Park, Korea, 2014,  survivor63 |  | 57.6  +/- 15.4 | 27 | 48 | 42 | 48 | NR | 19 | 31 +/- 17 | NR | - | - |
| Patricio,  Belgium, 201964 | 112  42 IHCA  70 OHCA | 54 +/-  16 | 83 | NR | 70 | 93 | 77 | 35 | <75 minutes low flow | 6 IHCA, 12 OHCA | N=6 | N=0 |
| Peigh, USA, 2015 non survivor65 | 23 IHCA (non survivor + survivor) | 45 +/-  16 | 6 | NR | 18 | 23 | NR | 6 | 57 +/- 35 | - | N=2 | N=5 |
| Peigh, USA, 2015  survivor65 |  | 46 +/-  10 | 9 | NR |  |  |  | 2 | 52 +/- 28 | NR | - | - |
| Pozzi, France, 201566 | 68 OHCA | 43.7  +/- 11.4 | (73.5%  which = 49.98) | 39.7%  which = 26.99 | 72.1% =  49.028 | 68 | 68 | 47.1% | No flow: 2.1 +/- 2.2  Low flow: 83.6  +/- 20.7 | 4.4% = 2.992 | N=6 | N=4 |
| Richardson, Australia/Fra nce, 201667 | 1796  (location not reported) | NR | 1224 | NR | NR | NR | NR | NR | NR | NR | N=0 | N=0 |
| Rousse, France, 2015  68 | 22 OHCA | 46.6  +/- 12 | 19 | NR | 22 | 22 | 22 | 13 | No flow < 5min Low flow: 106  +/- 16 | 1 | N=3 | N=3 |
| Ryu, Korea, 201569 | 115  96 IHCA  19 OHCA | 58 (45-  66) | 80 | 44 | NR | 115 | 9 | 48 | 34 (20-53) | 68 (61 IHCA  and 7 OHCA) | N=0 | N=0 |
| Sato, Japan, 201870 | 34 OHCA | 62  (56,66) | 30 | 25 | 26 | 28 | 19 | 23 | EMS: 61 (45-  74)  RRC: 52 (46-  59) | 3 | N=4 | N=3 |

| Schober, Austria, 201771 | 7 OHCA | 46 (31-  59) | 5 | 2 | 2 | 6 | 2 | 4 | no/low flow 97  (79-147)  admission to ECPR/ROSC: 55(45-68) | 1 | N=4 | N=2 |
| --- | --- | --- | --- | --- | --- | --- | --- | --- | --- | --- | --- | --- |
| Shin, Korea, 201372 | 85 IHCA | 59.9  +/- 15.3 | 53 | 38 | 79 | 85 | NR | 25 | NR | 22 | N=2 | N=8 |
| Siao, Taiwan, 201573 | 20  2 IHCA  18 OHCA | 54.55  +/- 11.94 | 18 | 13 | 16 | NR | 20 | 20 | Survivors: No flow: 1-4  Low flow: 56.5  +/- 62.02  No flow: 1-5 Low flow:41.5  +/- 25.73 | 8 (IHCA and OHCA) | N=4 | N=5 |
| Spangenberg  , Germany, 201874 | 60  22 IHCA  38 OHCA | NR | NR | 40 | 54 | 55 | 44 | 32 | Low flow 62.2  +/- 3.5 | 18 | N=2 | N=0 |
| Tanno, Japan, 200775 | 66 OHCA | 52.1  +/- 14.4 | 56 | NR | NR | 29 | 26 | 36 | NR | 7 | N=5 | N=3 |
| Terri, Canada, 201876 | 9  8 IHCA  1 OHCA | 52(45,  58) | 6 | 7 | 11 | 8 | 0 | 5 | 71.44 +/- 40.99 | 6 | N=1 | N=0 |
| Voicu, France, 201777 | 46  19 IHCA  27 OHCA | 56 (49-  62) | 38 | 36 | NR | 43 | 37 | 29 | 106 (72-132) | 2 IHCA, 2 OHCA | **N=1** | **N=5** |
| Wengemayer  , Germany, 201778 | 133  74 IHCA  59 OHCA | 58.7  +/- 2.6 | 74.4 % | NR | NR | 113 | NR | NR | No flow: 2.6 +/- 0.8  Low flow: 59.6  +/- 5 | NR | N=0 | N=3 |
| Yukawa,  Japan, 201779 | 79 OHCA | 59  (48.5-  64.5) | 65 | 39 | NR | 79 | 46 | 58 | 45 (40-56.5) | 11 | N=5 | N=1 |
| Yu, Taiwan, 2019 (18-  65)80 | 340  253 IHCA  87 OHCA | 49.4  +/- 11.8 | 269 | 118 | 230 | NR | NR | NR | 40.3 +/- 21.2 | 94 | N=1 | N=1 |
| Yu, Taiwan, 2019 (65-  75)80 | 93  78 IHCA  15 OHCA | 69.5  +/- 3 | 66 | 47 | 86 | NR | NR | NR | 41 +/- 18.6 | 23 | N=1 | N=1 |
| Zakhary, Australia, 201881 | 75  37 IHCA 38 OHCA | 50 (35-  59) | 61 | 33 | 51 | 31 | 36 | 43 | 58 (44-75) | 16 IHCA and 7 OHCA | N=6 | N=6 |

**Supplementary Table 4. Inclusion criteria frequency in prospective and retrospective studies.**

| **Inclusion criteria** | **No. of Prospective studies** | **% Prospective** | **Number of retrospective studies** | **% Retrospective** |
| --- | --- | --- | --- | --- |
| **age** | **11** | **85** | **30** | **73** |
| **initial shockable rhythm** | 6 | 46 | 7 | 17 |
| **cardioversion** | 2 | 15 | 1 | 2 |
| **estimated low flow time** | 6 | 46 | 12 | 29 |
| **bystander CPR** | 7 | 54 | 17 | 41 |
| **end tidal CO2 >10 mmHg** | 2 | 15 | 5 | 12 |
| **Pa O2 >50mmHg** | 2 | 15 | 1 | 2 |
| **lactic acid < 18 mmol/L** | 2 | 15 | 1 | 2 |
| **witnessed** | 9 | 69 | 16 | 39 |
| **refractory cardiac arrest** | 6 | 46 | 14 | 34 |
| **cardiac aetiology** | 7 | 54 | 9 | 22 |
| **mechanical CPR** | 4 | 31 | 0 | 0 |
| **non-shockable rhythm with reversible origin** | 1 | 8 | 0 | 0 |
| **mobile cardiothoracic team availability** | 1 | 8 | 0 | 0 |
| **absence of severe comorbidities** | 9 | 69 | 24 | 59 |
| **signs of life** | 1 | 8 | 0 | 0 |
| **absent bleeding** | 7 | 54 | 13 | 32 |
| **reversible cause including VF/VT** | 1 | 8 | 4 | 10 |
| **absence of ROSC** | 4 | 31 | 6 | 15 |
| **unconscious with dilated pupil during CPR** | 1 | 8 | 0 | 0 |
| **normothermic arrest** | 2 | 15 | 2 | 5 |
| **informed consent** | 0 | 0 | 7 | 17 |


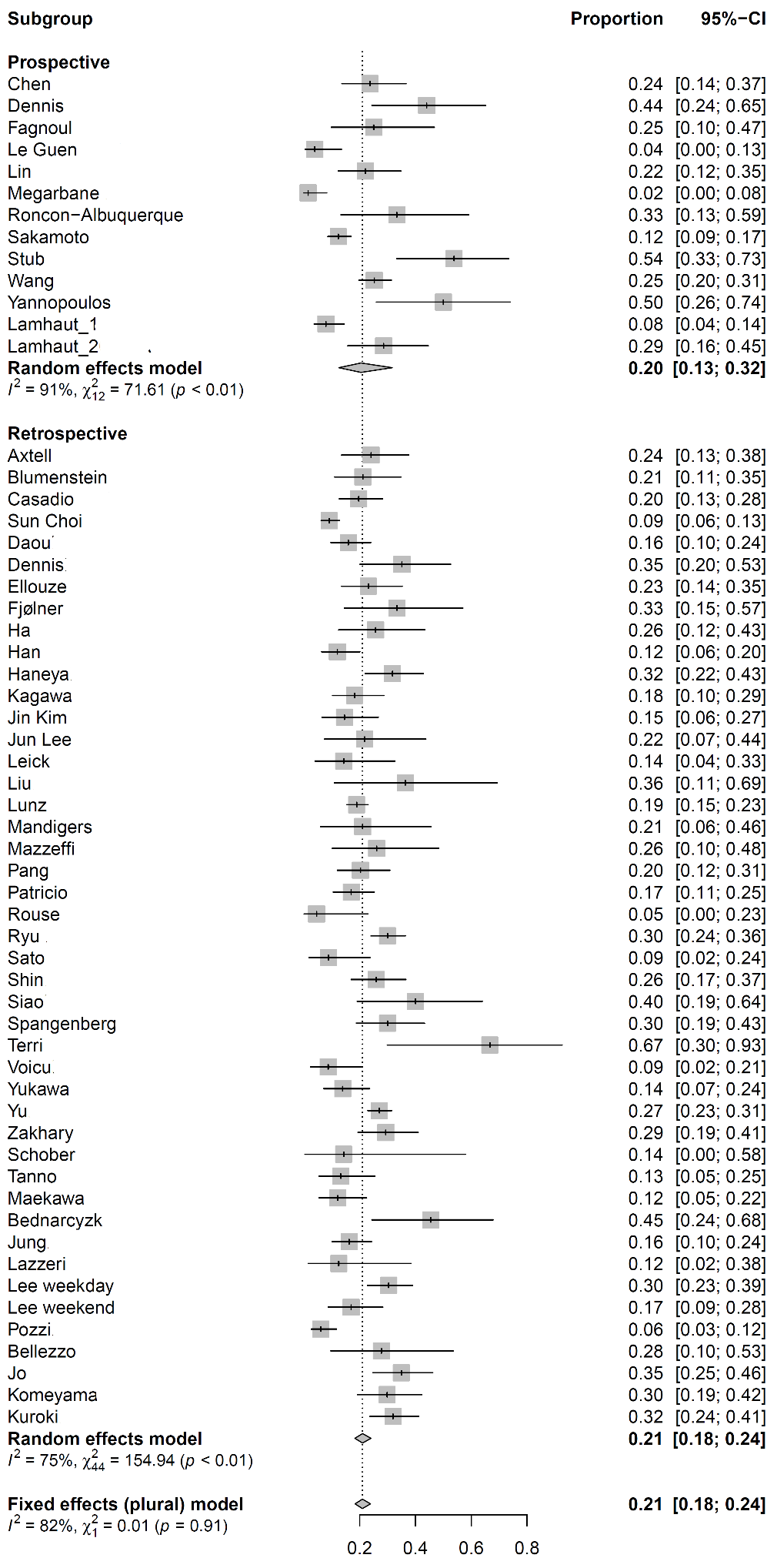
**Supplementary Figure 1:** Study specific forest plot of main findings
